# Imaging-Based Drug Penetration Profiling in an Excised Sheep Cornea Model

**DOI:** 10.3390/pharmaceutics16091126

**Published:** 2024-08-26

**Authors:** Karla Viehmeister, Aurélie Manuelli, Camille Guerin, Sebastian Kappes, Alf Lamprecht

**Affiliations:** 1 Department of Pharmaceutics, Institute of Pharmacy, University of Bonn, Gerhard-Domagk-Str. 3, 53121 Bonn, Germany; karla.viehmeister@uni-bonn.de (K.V.);; 2 Horus Pharma, 22, Allée Camille Muffat, Inedi 5, 06200 Nice, France; 3 Université de Franche-Comté, INSERM UMR1098 Right, 25030 Besançon, France

**Keywords:** sheep cornea, corneal penetration, corneal distribution, penetration kinetics, corneal viability, confocal laser scanning microscopy, depth cut, Franz diffusion cell, fluorescein sodium, hyaluronic acid

## Abstract

Formulations designed to address ocular conditions and diseases are predominantly administered topically. While in vitro test systems have been developed to assess corneal permeation under extended contact conditions, methods focusing on determining the penetration depth and kinetics of a substance within the cornea itself rather than through it, are scarce. This study introduces a method for time-dependent penetration depth analysis (10 and 60 min) by means of a semiquantitative imaging method in comparison with a quantitative corneal depth-cut technique, employing fluorescein sodium at concentrations of 0.2 and 0.4 mg/mL as a small molecule model substance and sheep cornea as a human surrogate. Excised tissues exhibited sustained viability in modified artificial aqueous humor and maintained thickness (746 ± 43 µm) and integrity (electrical resistance 488 ± 218 Ω∙cm^2^) under the experimental conditions. Both methods effectively demonstrated the expected concentration- and time-dependent depth of penetration of fluorescein sodium, displaying a significantly strong correlation. The traceability of the kinetic processes was validated with polysorbate 80, which acted as a penetration enhancer. Furthermore, the imaging-based method enabled detecting the retention of larger structures, such as hyaluronic acid and nanoemulsions from the commercial eyedrop formulation NEOVIS^®^ TOTAL multi, inside the lacrimal layer.

## 1. Introduction

Treatment of diseases and pathological conditions of the eye is typically conducted by the application of topical formulations, such as eye drops, creams, or ointments. However, the development of effective topical formulations for the eye remains a challenge due to tear flow, naso-lachrymal drainage, and blinking, which thoroughly clear the corneal surface of an active pharmaceutical ingredient (API) before effective doses penetrate and permeate the cornea, as the eye’s outermost barrier, to the designated target tissue [1]. Furthermore, the cornea itself effectively limits transport into the eye due to its multilayered structure [2,3]. It is composed of five distinct layers, namely the epithelium, Bowman’s layer, stroma, Descemet’s membrane, and endothelium, which contribute to its structural integrity, transparency, and critical barrier function. The multilayered epithelium of surface cells with tight junctions, wing cells, and basal columnar cells is particularly difficult for substances to overcome [4]. Although the corneal barrier could easily be bypassed by intravitreal injections, this form of application requires administration by health professionals, and thus, topically administered products retained the greatest market share of ophthalmologic products in 2022 [5]. To assess the corneal permeation of APIs from topical formulations in ophthalmic formulation development, various in vitro and ex vivo test systems have been established. For in vitro ocular drug delivery models, primary cell culture [6], immortalized cell lines [7,8], or reconstructed tissue culture [9,10] are being used.

However, 3D culture models do not provide full-thickness tissue layers, which can subsequently lead to a false overestimation of drug penetration [11]. Therefore, ex vivo permeation studies are conducted either as diffusion cell assemblies with the whole cornea as the diffusion membrane or as whole eye setups, both considering all corneal layers and allowing analysis of drug amounts after passage of the entire corneal tissue. Of course, the human cornea represents the gold standard in these studies, but as it is scarce and mainly reserved for transplantation, animal surrogate tissues from different species, such as rabbits or pigs, have been used [12]. Nevertheless, due to the excellent barrier properties, the comparably small corneal area present for diffusion in these animal tissues would either require specialized study setups and highly sensitive devices for quantification of trace amounts of permeant or a prolonged study time. Prolonging the duration of such ex vivo experiments not only leads to a vastly increased contact time of formulation to the tissue compared to in vivo conditions but also comes at the risk of reduced corneal integrity, accompanied by a decrease in viability and corneal swelling that might lead to overestimated permeation results [13]. Additionally, not every API that is topically applied for the treatment of eye diseases has its target site behind the corneal barrier. Instead, diseases like dry eye disease or keratitis are typical impairments of the cornea that rely on drug integration into the lacrimal film or penetration into the cornea rather than permeation through it [14]. In these cases, standard permeation experiments are less suitable since their focus lies on the amount delivered to the basolateral compartment. Accordingly, a detailed analysis of both substance retention on the eye’s surface and its residence time-dependent distribution into the cornea is needed in formulation development, yielding conclusive results in a suitable timeframe under conditions that ensure corneal integrity to better mimic in vivo conditions. To this end, we present an adaptation of the Franz diffusion cell permeation setup, directly analyzing the penetration of substances into the corneal tissue and the distribution therein using an image-based approach. The sheep cornea, which displays all layers found in the human cornea (Figure S1), was used as a surrogate [15,16]. The total corneal thickness of sheep, pig, and rabbit corneas is similar to that of humans, but the relative epithelial thickness is more analogous to the human cornea in sheep and rabbits [11,17,18,19,20]. The larger corneal area of sheep compared to pigs and rabbits offers improved handling and analysis options, making it the most suitable surrogate for the presented study [11]. The experimental conditions were optimized to ensure prolonged tissue viability and integrity over the duration of the experiments. The model was established by analyzing the time- and concentration-dependent penetration kinetics of the small model molecule fluorescein sodium (FS), which has broadly been used in eye research [21,22,23,24,25], as well as the influence of polysorbate 80 (P80) on FS-penetration semi-quantitatively by confocal laser scanning microscopy (CLSM). The image-derived results were then confirmed by quantitative analysis of extracted FS from corneal depth cuts. Moreover, the applicability of the image-based technique to more complex formulations containing larger molecules was demonstrated by analyzing an eyedrop nanoemulsion formulation inspired by the composition of the lacrimimetic commercial product NEOVIS^®^ TOTAL multi and subsequent assessment of the penetration of its key components or the adhesion to surface cells, i.e., macromolecular hyaluronic acid and the lipid emulsion droplets.

## 2. Materials and Methods

### 2.1. Materials

Dimethylthiazolyldiphenyl-tetrazolium bromide (MTT) and 2-[4-(2-hydroxyethyl) piperazin-1-yl]ethanesulfonic acid (HEPES) were purchased from Carl Roth GmbH + Co.KG (Karlsruhe, Germany). Dimethylsulfoxide (DMSO) was obtained from VWR International (Radnor, PA, USA). Sodium dodecylsulfate (SDS) was purchased from Caesar & Loretz GmbH (Hilden, Germany). Triton^®^ X-100, Ethylendiamine, N-ethyl-N9-(3-dimethyl-aminopropyl) carbodiimide, Fluorescein-5(6)-isothiocyanate (FITC), and 1,1′-Dioctadecyl-3,3,3′,3′-tetramethyl-indocarbocyanine perchlorate (DiI) were obtained from Sigma Aldrich (St. Louis, MO, USA). Dulbecco’s minimum essential medium (DMEM) and penicillin plus streptomycin were purchased from Biochrom (Berlin, Germany). Fluorescein sodium was supplied by Merck KGaA (Darmstadt, Germany). Montanox™ 80 PPI (polysorbate 80 for injection) was a gift from Seppic (La Garenne Colombes, France). Sodium hyaluronate (HA), Hydroxypropylmethylcellulose (HPMC), Miglyol 812, and Soybean lecithin lipoid p75 were kindly provided by Horus Pharma (Nice, France). All chemicals used to prepare buffers were of analytical grade or higher. Milli-Q^®^ water (Q-Gard^®^ Purification Catridge, Type 1 water) was used throughout the experiments. Sheep heads of class A sheep (Regulation (EU) No. 1308/2013), breed: Einola, 4–6 months old, following the routine slaughtering procedure, were purchased from a local commercial slaughterhouse (Regulation (EU) No. 1069/2009, category three material (article 10a) used for scientific purposes (article 16f.).

### 2.2. Methods

#### 2.2.1. Cornea Acquisition

On the day of slaughter for human consumption, sheep heads were obtained freshly prior to each experiment, with eyes closed during transport. The cornea with an adjacent scleral ring of about 3–5 mm was carefully dissected from the eyeball, the iris cautiously removed, and the cornea directly placed in modified artificial aqueous humor (mAAH) until immediate use in the respective experiment. The mAAH was composed of NaCl 130 mM, KCl 5 mM, CaCl_2_ 1 mM, MgCl_2_ 1 mM, D-Glucose 2.5 mM, NaHCO_3_ 10 mM, HEPES 10 mM, and NaN_3_ 3.08 mM (pH 7.3) in accordance with the artificial aqueous humor (AAH) of Linetsky et al. that does not contain sodium azide [26]. Opaque corneas indicating tissue damage were discarded. Experimental replicates were always taken from different sheep heads.

#### 2.2.2. Corneal Characterization

Viability: Corneal viability was assessed by measuring tetrazolium reductase activity. Freshly excised cornea without any adjacent tissue was quartered, weighed, covered with 2 mL of test medium, and incubated for 24 h at 37 ± 0.5 °C. Afterward, the test medium was carefully removed, tissue pieces were washed with mAAH, and 2 mL of MTT reagent solution (1 mg/mL in mAAH) was added and incubated for 2 h at 37 ± 0.5 °C. The tissue pieces were washed and transferred into microtubes individually. Formazane was extracted with 1.5 mL of DMSO containing 5% SDS and 1% HCl (1M) in an ultrasound bath. After centrifugation, supernatant absorption was detected at 560 nm with an EnSpire Multimode Plate Reader (PerkinElmer, Inc., Shelton, CT, USA). Freshly excised cornea without further media treatment was used as a positive control, whereas cornea treated with Triton^®^ X-100 2.5% for 24 h served as a negative control. Absorption results were normalized to the tissue weight. Three corneas from different sheep were used for each treatment, and mean viability was calculated as a percentage of the positive control after subtracting the negative control. Tested media were phosphate-buffered saline (PBS), DMEM, HEPES buffer (20 mM), AAH, AAH with penicillin and streptomycin (AAH_PS_) at concentrations of 100 U/mL and 100 µg/mL, respectively, and mAAH.

Dimensions and Thickness: Corneal surface dimensions were measured via image analysis with Fiji 2.1.0 software [27]. For thickness acquisition, corneas were incubated in mAAH with and without P80 0.25% (*m*/*v*) at 35 ± 1 °C for 6 h, recording the thickness with a digital indicator (Mitutoyo ID-125B) at timepoints 0, 10, 30, and 60 min and hourly thereafter.

Electrical Resistance: Corneas were clamped in Franz diffusion cells (FDC) and maintained at 35 ± 1 °C surface temperature. Corneal electrical resistance was measured with an LCR meter (Escort Instruments Dual Display LCR Meter ELC-131D) at a test frequency of 120 Hz with two electrodes in contact with mAAH or mAAH + P80 0.25% (*m*/*v*) on the epithelial side and mAAH on the endothelial side at timepoints 0, 10, 30, and 60 min and hourly thereafter for a total duration of 6 h. Electrodes did not touch the corneal surface to avoid physical damage and were kept at the same distance for each measurement. Resistance values were reduced by the blank value (FDC filled with mAAH lacking a cornea as a membrane) and normalized to the area.

#### 2.2.3. Preparation of Tested Formulations

Fluorescein Sodium Donor Solutions: For depth penetration analysis, FS solutions were freshly prepared prior to each experiment by dissolving FS in the respective solvent with stirring and in the absence of light until a clear solution was obtained. FS penetration was compared at concentrations of 0.2 and 0.4 mg/mL in mAAH (FS 0.2 and FS 0.4) and with the addition of P80 0.25% (*m*/*v*) (FS 0.2 P80).

Commercial Eyedrop Formulation: The tested commercial eyedrop formulation was NEOVIS^®^ TOTAL multi, which is a nanoemulsion containing HA and lipids. It was replicated to fluorescently label the ingredients of interest during the preparation. HA was labeled with FITC in a subsequent process of final eyedrop preparation. Lipid phase labeling with DiI took place in a pre-step of the nanoemulsion preparation.

Labeling Process for Sodium Hyaluronate: HA was labeled with FITC in a process modified from Liberda et al. [28], where the carboxylic acid groups were derivatized to carboxamide with ethylendiamine and N-ethyl-N9-(3-dimethyl-aminopropyl) carbodiimide, subsequently coupled with FITC, purified via dialysis (MWCO 50 kDa), and lyophilized to the final product FITC-HA.

Preparation of Labelled Lipid Phase: To replicate the lipid phase (LP) of NEOVIS^®^ TOTAL multi-eye drop formulation, Miglyol 812 N and Soybean Lecithin Lipoid P75 were weighed into a SnapCap vial, and 200 μL of DiI solution (1 mg/mL in DCM) was added. Excess DCM was evaporated, and preheated water (60 °C) was added and stirred at 40 °C for 30 min. The mixture was homogenized by an ultrasonic probe (Sonopuls HD 2200 with TT13, Bandelin, Berlin, Germany) for 3 min at 51% amplitude. After cooling, water was added to achieve the labeled lipid phase (DiI-LP) as a concentrated pre-nanoemulsion. Particle size (Z-average) was analyzed by Photon Correlation Spectroscopy (PCS) using the Nanopartica SZ-100 (Horiba) at a 173° angle.

Final NEOVIS^®^TOTAL multi Replica: The aqueous phase was composed of (FITC-)HA, HPMC, sodium citrate, citric acid, sodium chloride, and water. An equal volume mixture of aqueous phase and pre-nanoemulsion was equivalent to the NEOVIS^®^ TOTAL multi-eye drop formulation. To minimize crosstalk in fluorescence analysis, two NEOVIS^®^ TOTAL multi-equivalent formulations were prepared, one with FITC-HA in the aqueous phase and non-labeled LP and another with non-labeled HA in the aqueous phase and DiI-LP. A non-labeled formulation of NEOVIS^®^ TOTAL multi served as a control.

#### 2.2.4. Penetration Assay and Analysis

Cornea Preparation: Corneas were clamped in the FDC with the epithelium facing upwards with an area of 0.785 cm^2^ and equilibrated to a surface temperature of 35 ± 1 °C. The receptor compartment of 5.5 mL volume was filled with mAAH. Corneas were incubated with respective donors for 10 and 60 min. After incubation, the epithelium was washed with mAAH and processed for depth imaging analysis and, in the case of FS solutions, additionally for depth-cut analysis (Figure S2). The comparison of different FS concentrations and the impact of the potentially penetration-influencing additive P80 was performed in separate experimental setups.

Depth Image Processing and Analysis: Corneal cross sections of 50 μm in the case of FS-incubated corneas and 30 µm for the NEOVIS^®^ TOTAL multi replica were cut from posterior to anterior with a MEV Cryostat (SLEE medical GmbH, Germany). Fluorescence images were acquired via CLSM (Nikon, Japan). Z-stacks were analyzed using Fiji software [27]. Depth imaging profiles were obtained by plotting the fluorescence intensity as a gray value over distance from the epithelial surface.

Depth-Cut Processing and Analysis: For depth-cut analysis, three biopsy punches (Ø 4 mm) were taken from the corneal incubation area, placed in a Tissue-Tek^®^ Cryomold^®^ (15 × 15 × 5 mm) epithelium facing downwards, embedded in Cryomatrix™ (Epredia, Breda, The Netherlands), and immediately frozen. Depth cuts of 40 µm parallel to the epithelium were taken with a cryostat and placed in microtubes. FS was extracted with 500 µL of water on an orbital shaker, followed by fluorescence analysis with the EnSpire Multimode Plate Reader (PerkinElmer, Inc., Shelton, CT, USA). Depth-cut profiles were obtained by calculating FS mass per area in a defined depth range. To assess the correlation between the gray value of the depth imaging profiles and mass per area of the depth-cut profiles, the mean gray value in the respective depth range of 40 µm was calculated, and the Pearson correlation coefficient was computed with GraphPad Prism 8.0.2 software.

#### 2.2.5. Corneal 3D Imaging

For 3D imaging, corneas were clamped in FDC as described above. To stain upper epithelial nuclei, a solution of Hoechst 33342 (10 µg/mL) was apically applied for 20 min. After washing the epithelium with mAAH, the NEOVIS^®^ TOTAL multi-eyedrop formulation was applied for 60 min. Three-dimensional imaging was performed on CLSM.

#### 2.2.6. Statistics

Data are presented as mean ± standard deviation unless otherwise stated. GraphPad Prism 8 software was used to test for correlation and statistical significance. Different tests were performed depending on the sample size in each group, whether multiple comparisons had to be considered, and whether the groups had the same standard deviation. The statistical tests and significance levels are marked accordingly in the respective sections of the results.

## 3. Results

### 3.1. Corneal Characterization

#### 3.1.1. Viability

**Figure 1:**
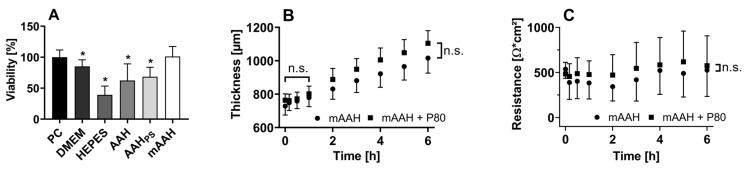
(**A**) Corneal viability after incubation with different media for 24 h as a percentage of the positive control (PC). Tested media were Dulbecco’s minimum essential medium (DMEM), HEPES buffer (20 mM), artificial aqueous humor (AAH), AAH with penicillin/streptomycin (AAH_PS_), and modified AAH (mAAH). Statistical analysis for viability was performed as multiple comparisons (Brown–Forsythe and Welch ANOVA tests), * *p* < 0.05 vs. PC. (**B**) Corneal thickness during incubation in mAAH and mAAH + P80 0.25% (mAAH+P80). (**C**) Corneal resistance in an FDC setup with mAAH or mAAH+P80 as a donor. Statistical analysis (multiple *t*-tests using the Holm–Sidak method) showed no significant differences in thickness or electrical resistance between the incubation/donor media at any timepoint (α = 0.05). Multiple comparison analyses with ordinary one-way ANOVA corrected with the Tukey test resulted in no significant difference in thickness within one hour of measuring and no significant difference in resistance within 6 h of measuring (*p* < 0.05).

The untreated cornea measured immediately after dissection (positive control) and the negative control showed a UV absorbance per tissue weight of 11.22 ± 1.31 g^−1^ and 0.08 ± 0.15 g^−1^, respectively, in the colorimetric test. While viability decreased significantly over 24 h in DMEM, HEPES buffer 20 mM, AAH, and AAH with penicillin/streptomycin (AAH_PS_) compared with the positive control (*p* < 0.05), initial corneal viability was maintained in mAAH and, therefore, used in all subsequent experiments (Figure 1A). The viability results of corneas incubated with PBS are not included since PBS caused visible stromal swelling, opacity, and damage to the epithelium.

#### 3.1.2. Dimensions and Thickness

The surface dimensions of the sheep corneas used in this study were 14.1 ± 1.1 mm and 19.7 ± 0.9 mm in height and width, respectively. The corneal thickness was 746 ± 43 µm initially and did not change significantly (*p* < 0.05) within one hour regardless of the incubation medium—mAAH or mAAH + P80 0.25% (*m*/*v*). Corneas swelled to a thickness of 1064 ± 86 µm after 6 h of incubation (Figure 1B).

#### 3.1.3. Electrical Resistance

The mean electrical resistance of the corneal tissue within the FDC setup was 488 ± 218 Ω∙cm^2^ and did not vary significantly during the 6 h observation (*p* < 0.05). The addition of P80 0.25% (*m*/*v*) to mAAH had no significant influence (*p* < 0.05) on the corneal resistance (Figure 1C).

### 3.2. Penetration Analysis

#### 3.2.1. Small Molecule—Fluorescein Sodium

**Figure 2:**
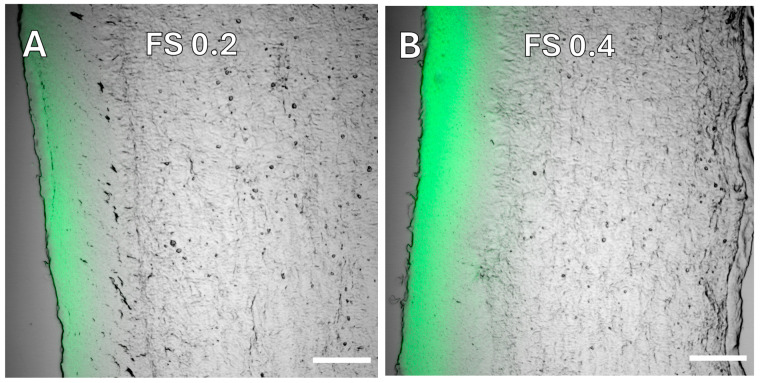
Confocal laser scanning microscopy. Cross sections of sheep cornea were incubated with FS-solution (**A**) at 0.2 mg/mL (FS 0.2) and (**B**) at 0.4 mg/mL (FS 0.4) for 60 min. Layered image of transmission light and laser scan (488 nm). The apical direction points left. The scale bar is 200 µm.

Exemplary images of corneal cross sections after 60 min incubation with the compared FS donor concentrations (0.2 and 0.4 mg/mL) show fluorescence intensity and depth penetration increase with higher FS content (Figure 2).

**Figure 3:**
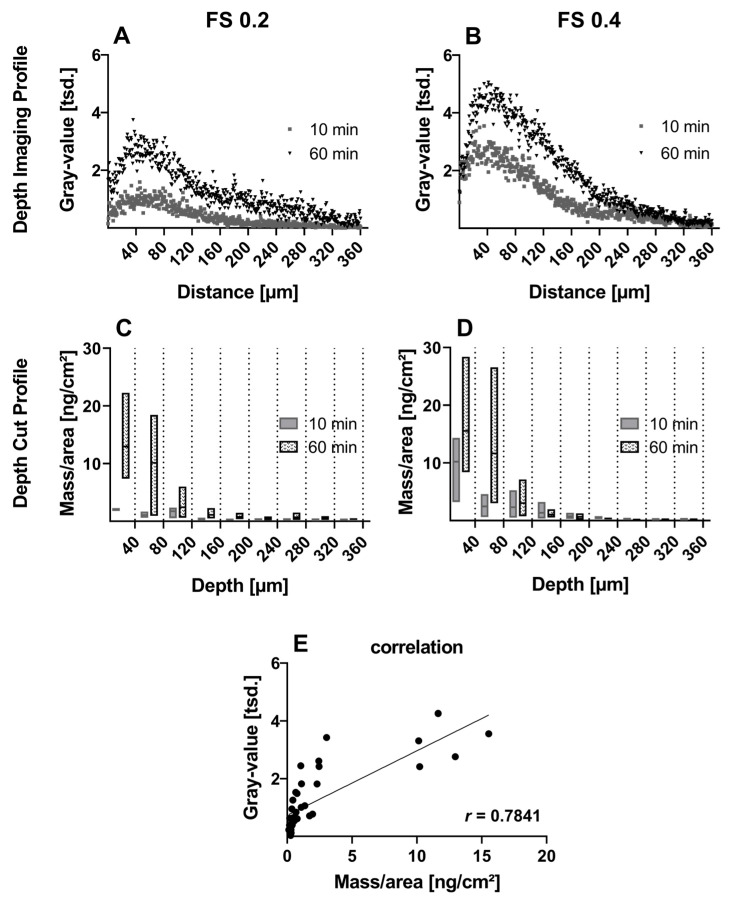
(**A**,**B**) Depth imaging profiles and (**C**,**D**) depth-cut profiles after incubation with fluorescein sodium solution at concentrations of 0.2 and 0.4 mg/mL (FS 0.2, FS 0.4) for 10 and 60 min. Depth imaging profiles are presented as means for clarity reasons. Depth-cut profiles are presented as mass/area in the respective previous depth range of 40 µm. (**E**) Correlation of gray value and mass/area from both concentrations and both incubation times with Pearson correlation coefficient r (*p* < 0.05) and reference line of regression.

The profile plot analysis with Fiji software [27] from apical to basolateral direction for both incubation times (10 and 60 min) and concentrations results in the respective depth imaging profiles (Figure 3A,B). They present a time- and concentration-dependent FS penetration increase into the cornea. The respective depth-cut profiles confirm these findings. The effect is especially prominent for the 10 min incubation time, where the maximum fluorescence signal at 40 µm depth in the depth image profiles doubled with higher FS concentration (Figure 3A,B), and accordingly, the FS mass/area is increased in the first 40 µm depth range from FS 0.2 to FS 0.4 (Figure 3C,D). The Pearson correlation of the gray value from the depth imaging profile and the mass/area from the depth-cut profiles of both concentrations and incubation times shows a significantly strong positive correlation (r(36) = 0.7841, *p* < 0.05) (Figure 3E).

**Figure 4:**
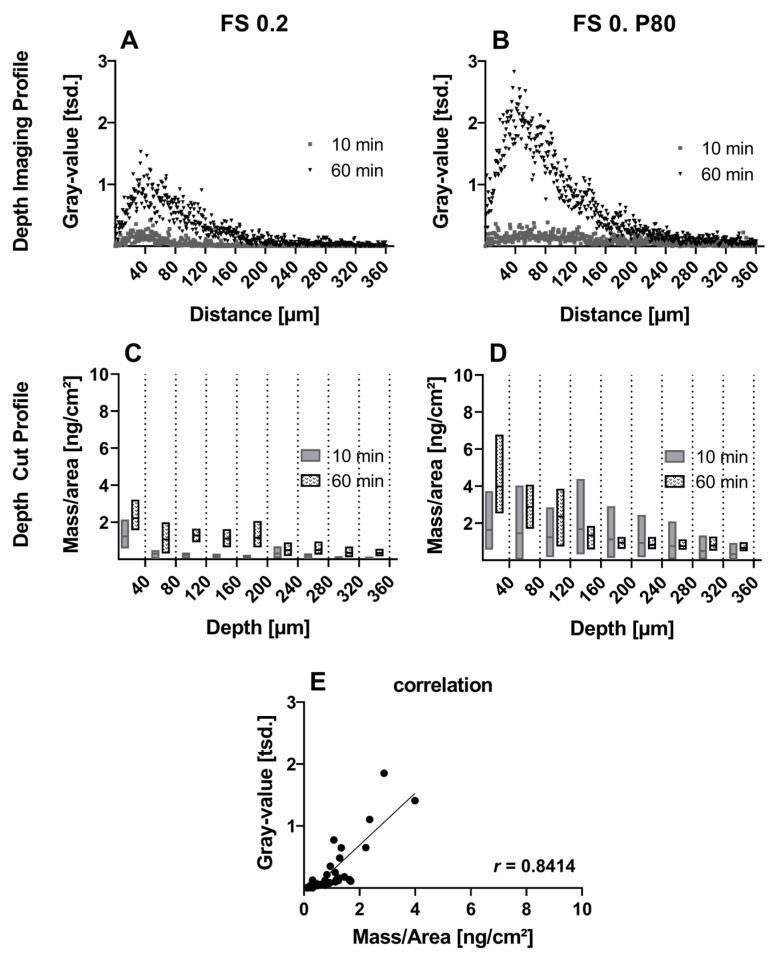
(**A**,**B**) Depth imaging profiles and (**C**,**D**) depth-cut profiles after incubation with fluorescein sodium solution at concentration 0.2 mg/mL (FS 0.2) and with the addition of polysorbate 80 0.25% (FS 0.2 P80) for 10 and 60 min. Depth imaging profiles are presented as means for clarity reasons. Depth-cut profiles are presented as mass/area in the respective previous depth range of 40 µm. (**E**) Correlation of gray value and mass/area from both formulations and both incubation times with Pearson correlation coefficient r (*p* < 0.05) and reference line of regression.

The addition of P80 in FS 0.2 compared to FS 0.2 solution leads to an increase in FS penetration depth, with the addition of P80 shown primarily in the depth-cut profiles (Figure 4C,D). A time-dependent increase in penetration depth is shown for both donor solutions and incubation times (Figure 4). The Pearson correlation coefficient of gray value and mass/area is significantly strong positive (r(36) = 0.8414, *p* < 0.05) (Figure 4E).

#### 3.2.2. Macromolecules—Hyaluronic Acid—And Lipids from Eyedrop Formulation

The depth imaging technique was expanded to include macromolecules such as HA and lipids found in the commercial ocular formulation, NEOVIS^®^ TOTAL multi. Given their natural similarity to corneal components, HA and lipids were labeled with fluorescent markers. The covalent linkage of FITC to HA resulted in a significant fluorescence signal compared to unlabeled HA (*p* < 0.05). Particle size analysis via PCS of the pre-nanoemulsion showed a Z-average of 229.3 ± 4.3 nm (PDI 0.292 ± 0.051) for DiI-LP and 211.8 ± 2.0 nm (PDI 0.188 ± 0.027) for non-labeled LP.

**Figure 5:**
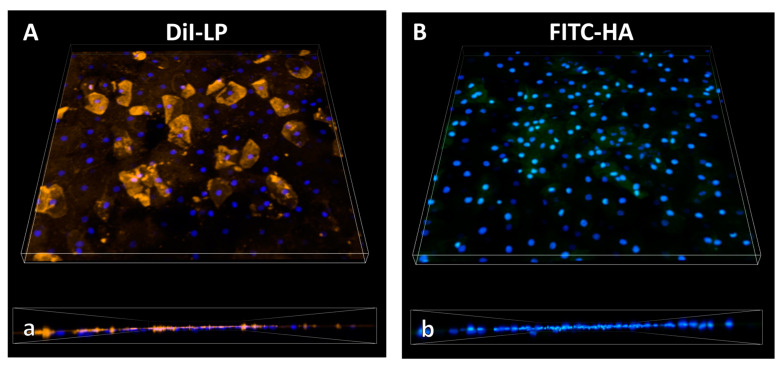
Confocal laser scanning microscopy. Three-dimensional images of sheep cornea incubated for 30 min with NEOVIS^®^ TOTAL multi replica containing (**A**,**a**) DiI-LP (yellow) and (**B**,**b**) FITC-HA (green) in XYZ− (upper row) and Y+-direction (bottom row). Upper epithelial nuclei are stained with Hoechst 33342 (blue). X—636.40 µm, Y—636.40 µm, Z—30.10 µm.

Penetration Analysis and Imaging of Eyedrop Formulation Replica: Three-dimensional imaging by CLSM of the fluorescent NEOVIS^®^ TOTAL multi-formulation replica applied to the cornea revealed the accumulation of LP on the surface, especially on and in dead surface cells, as indicated by large yellow regions on the corneal surface (Figure 5A). Deeper penetration of LP into the epithelium was not evident, as the DiI signal below the uppermost cell layer, displayed by the Hoechst-stained nuclei (blue), was absent (Figure 5a). Similar results were obtained for HA. The macromolecule adhered to surface cells, particularly visible in the center of the presented exemplary image section (Figure 5B), and did not penetrate below the first epithelial cell layer (Figure 5b).

**Figure 6:**
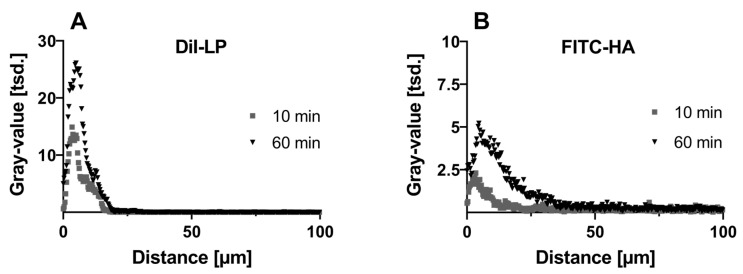
Depth imaging profiles of (**A**) lipid phase (DiI-LP) and (**B**) hyaluronic acid (FITC-HA) from NEOVIS^®^ TOTAL multi after incubation for 10 and 60 min. Depth imaging profiles are presented as means for clarity reasons.

Depth image processing and analysis were conducted to further analyze the fluorescence signal of DiI-LP and FITC-HA from NEOVIS^®^ TOTAL multi-analogous formulations after 10 and 60 min of corneal incubation. The penetration depth of 20 µm of the LP was similar for both incubation times. The amount of adhered LP increased with time, as indicated by the risen gray value after 60 min (Figure 6A). FITC-HA penetrated to a maximum depth of 25 µm after 10 min and increased to 40 µm after 60 min of incubation. Additionally, the gray value increased with time and thus the amount of HA (Figure 6B). Examples of the resulting CLSM images at each incubation timepoint are depicted in the Supplementary Materials (Figure S3).

## 4. Discussion

In this study, we established an ex vivo method to perform kinetic studies of small and macromolecular penetration into excised sheep corneas, especially in view of detailed analyses of the intracorneal compound distribution. The utilization of a vertical Franz diffusion cell setup was chosen due to its seamless donor and receptor compartment separation while maintaining physiological temperature. Although PBS is often used as receptor fluid for permeation analysis in similar diffusion cell assemblies [29,30], viability and integrity were not provided for sheep corneas in PBS. HEPES buffer was unable to sustain sheep corneal viability over 24 h, likely due to insufficient electrolytes and glucose. In contrast, DMEM and AAH variations, containing essential nutrients, showed higher viability. The superior preservation of corneal viability observed with mAAH may be ascribed to the preservative properties of sodium azide, which effectively inhibit not only bacterial growth, such as that inhibited by penicillin/streptomycin, but also fungal proliferation [31]. The surface dimensions and thickness of the chosen sheep breed are in accordance with previously reported work [15,32]. As this study focuses on depth penetration, it was crucial to consider corneal swelling. Prior analyses have investigated swelling in the cornea, especially in the stroma [33,34]. The selected one-hour experimental timeframe avoids any overlap with corneal swelling, thus preventing potential data distortion. Studies examining sheep corneal electrical resistance within diffusion cell set ups were not reported before. Our findings are in a similar order of magnitude to the electrical resistances of the cornea in other animal species, e.g., rabbit cornea (approximately 1000 Ω∙cm^2^ [35]) and pig cornea (871 ± 422 Ω∙cm^2^ [36], 606 ± 199 Ω∙cm^2^ [7]). Additionally, the sheep corneal resistance value (488 ± 218 Ω∙cm^2^) exceeded the lower limits of 400 Ω∙cm^2^ set for human corneal epithelial cell culture models with HCE-T cell lines [37]. Sheep cornea was hence confirmed as a suitable surrogate for the presented ex vivo kinetic study.

Drug permeation studies through the cornea are numerous, whereas methods to assess permeation and penetration kinetics within the cornea are rare. Mun et al. presented an image analysis of FS penetration in bovine cornea by differentiating the overcoming of the epithelium depending on β-cyclodextrin addition without further depth kinetic analysis [23]. Earlier studies established a method for analyzing the permeation kinetics of fluorophores in the rabbit cornea, which was further elaborated where the partitioning of the model compounds FS (hydrophilic) and rhodamine B (lipophilic) was non-destructively analyzed within the layers of the cornea with a custom-built confocal scanning microfluorometer (CSMF) [24,25,38]. The results were assessed after pseudo-equilibration and presented as fluorescence intensity over depth. However, results for epithelial incubation could only be provided for rhodamine B, where the equilibration time was rather short, and kinetic studies for FS were presented from the endothelial side.

The present study confirmed that FS does not penetrate through the cornea from the epithelial side to detect its fluorescent signal in sufficient amounts within all corneal layers. Nonetheless, this study unveiled two approaches to investigating the penetration kinetic processes of FS as a small hydrophilic model molecule. The image-based method showed the expected time- and concentration-dependent increase in FS penetration depth. Feasibility was further demonstrated by the addition of polysorbate 80, which is a polyoxyethylated nonionic surfactant reported to fluidize cell membranes and increase apical-to-basolateral permeability [39]. Polysorbate 80, therefore, likewise increased FS penetration depth in image and software analysis. The additional quantitative depth-cut method provided complementary results for both experimental setups (FS 0.2 vs. FS 0.4 and FS 0.2 vs. FS 0.2 P80), with a significantly strong correlation (r(36) > 0.78, *p* < 0.05) between the fluorescence signal (gray value) in image-based analysis and mass/area in depth-cut analysis in each experimental setup. While the reproducibility of FS 0.2 depth imaging and depth-cut results appear to be extensible, the strong correlation suggests the underlying cause may lie not in the individual methods but rather in the broader spectrum of corneal explant diversity. Therefore, an effective approach could involve increasing the number of corneas to address this variability. Nonetheless, since the image-based method is, as reported in previous studies [23,24,25,38], limited to fluorescent molecules, the depth-cut method offers the advantage of being potentially applicable to every molecule that penetrates in sufficient amounts into the cornea with individually adapted extraction and analysis.

It must be acknowledged that distinguishing every penetrating molecule from the constituents of the cornea poses a challenge. Although small drug molecules like FS can typically be separated from the tissue with ease, complex separation processes and analyses are required for molecules such as the glycosaminoglycan hyaluronic acid, which is intrinsically integrated into the stromal structure [40]. Thus, leveraging fluorescent labeling of the target ingredient remains advantageous for visualizing its kinetics within the cornea. Therefore, the image-based analysis was applied to examine the penetration depth of the macromolecule hyaluronic acid and lipid nanoparticles in the eyedrop formulation NEOVIS^®^ TOTAL multi. Both compounds are established for the treatment of dry eye disease (DED) [41,42]. The image analysis confirmed the absence of either HA or lipid phase inside the epithelium but showed an increase in signal intensity with incubation time for both, suggesting adhesion and integration of HA onto the corneal surface. The results for HA are in coherence with findings from other reports, which applied the described CSMF method to FITC-labeled chitosan–dextran sulfate nanoparticles with a particle size of 425.1 ± 3.2 nm [43]. Particles of such dimensions proved unable to overcome epithelial structures within 6 h. Only by removing the epithelium, kinetics within the stroma could be achieved [43]. The HA contained in the eyedrop formulation is a macromolecule, and therefore, penetration through the epithelium is not to be expected. The image-based method yielded heightened and marginally deeper fluorescence signals over time, suggesting an increased influx of HA and nanolipid droplets through and into the uppermost cell layer. The observations can underscore the lacrimimetic efficacy inherent in the formulation designed with endogenous components for the treatment of DED, a characteristic that can be empirically substantiated through the employed ex vivo method.

When transferring the image-based method to other formulations during their development, it must be considered that it is limited to fluorescent or fluorescently labeled ingredients of interest. Labeling non-fluorescent small molecules can surely alter their size and properties, affecting diffusion. For such molecules, the depth-cut approach may be more suitable. Additionally, when working image-based, the number and selection of images have to be chosen carefully, requiring a careful balance between time investment and representativeness; similarly, for the depth-cut method, the thickness of the cuts can be adjusted, demanding a compromise between precision and practicality. Despite these limitations, our methods are valuable for formulation development, providing precise and insightful analysis in a short timeframe.

## 5. Conclusions

The sheep cornea of the Einola breed was successfully implemented for assessing corneal API penetration ex vivo, and optimal experimental conditions were established regarding viability and integrity. The feasibility of penetration depth analysis in the cornea at relevant residence times via image fluorescence analysis for comparison of different formulations of FS was demonstrated and validated by depth-cut analysis. While the correlation of the two methods confirmed their comparable outcomes, the CLSM imaging-based method showed its potential and simplicity in the analysis of eyedrop formulation that efficiently retained at and integrated into the corneal surface, as illustrated with NEOVIS^®^ TOTAL multi, unambiguously locating hyaluronic acid and lipids on the apical surface of the tissue without deeper penetration, building the basis for the suggested lacrimimetic behavior of the formulation.

## Data Availability

Data are contained within the article and Supplementary Materials.

## Supplementary Materials

The following supporting information can be downloaded at: https://www.mdpi.com/article/10.3390/pharmaceutics16091126/s1, Figure S1. Structural comparison of schematic human corneas and microscopic images of sheep corneas. The middle image shows a PAS-stained cross-section of a sheep cornea. The right image shows sheep corneal epithelium—nuclei stained with Hoechst 33342 (blue) and membranes stained with WGA-Alexa647 (red) (created with BioRender.com). Figure S2: Schematic image of preparation, processing, and analysis in corneal penetration analysis via depth imaging and depth cut; Figure S3. Confocal laser scanning microscopy. Cross sections of blank sheep cornea in transmission light and at excitation with 543.5 nm and 488 nm laser (laser scan and layered image of transmission light and laser scan) for DiI (yellow) and FITC (green), respectively, after incubation with NEOVIS^®^ TOTAL multi (DiI-LP) and NEOVIS^®^ TOTAL multi (FITC-HA) at 10 and 60 min. The red indicators point to the basal membrane of the corneal epithelium. The apical direction points left. The scale bar is 200 µm.
