# Supplementary material for: Imaging-Based Drug Penetration Profiling in an Excised Sheep Cornea Model
Source: Pharmaceutics. 2024 Aug 26;16(9):1126. doi: 10.3390/pharmaceutics16091126 (PMC11435002; doi:10.3390/pharmaceutics16091126)
Supplement: Supplementary file 1 [file pharmaceutics-16-01126-s001.zip › pharmaceutics-3108263-supplementary.pdf]

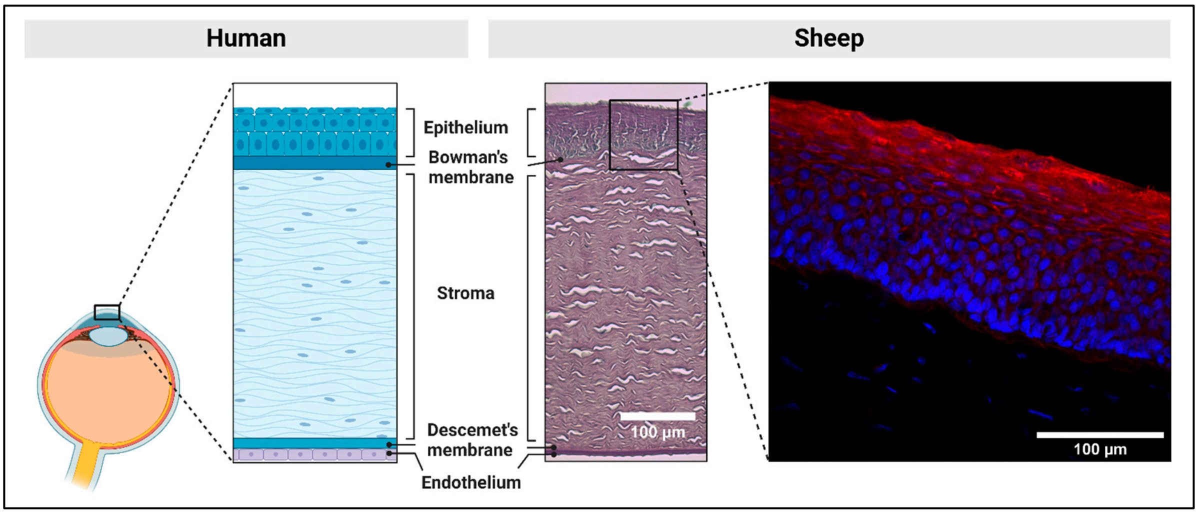

Figure S1. Structural comparison of schematic human cornea and microscopic images of sheep cornea. The middle image shows PAS-stained cross-section of sheep cornea. The right image shows sheep corneal epithelium – nuclei are stained with Hoechst 33342 (blue) and membranes are stained with WGA-Alexa647 (red). (Created with BioRender.com)

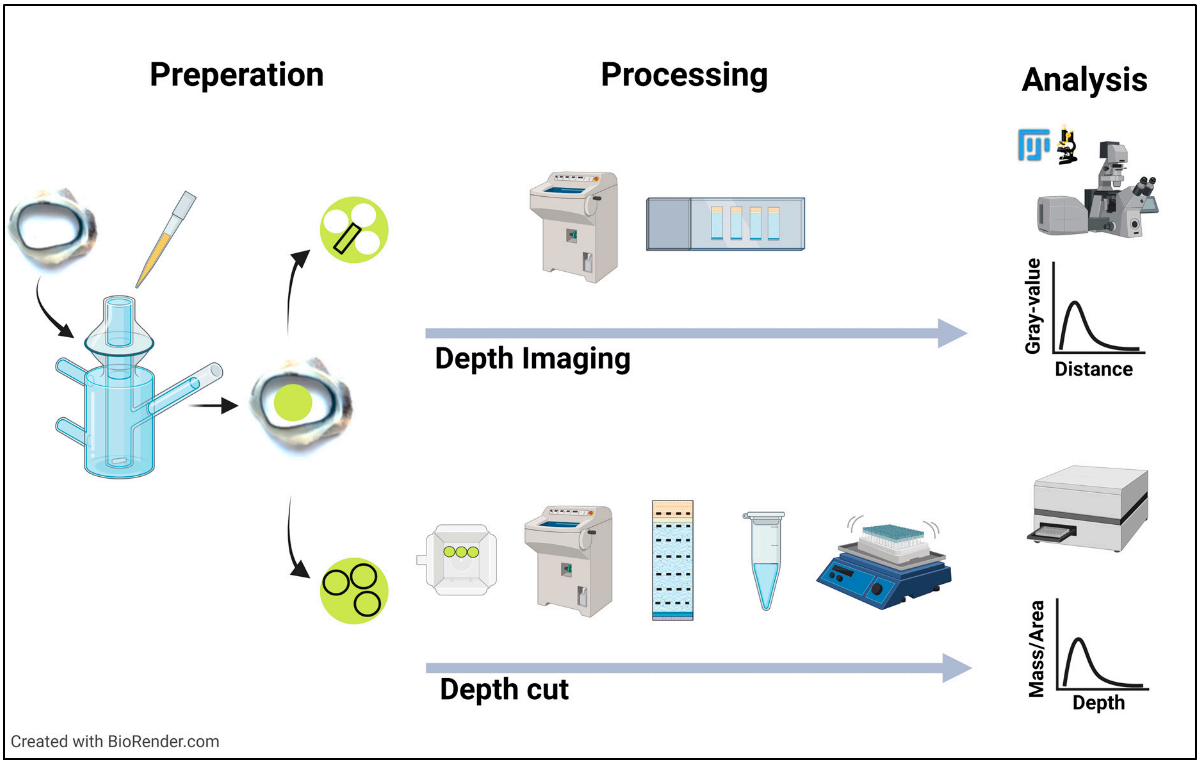

Figure S2. Schematic image of preparation, processing, and analysis in corneal penetration analysis via depth imaging and depth cut.

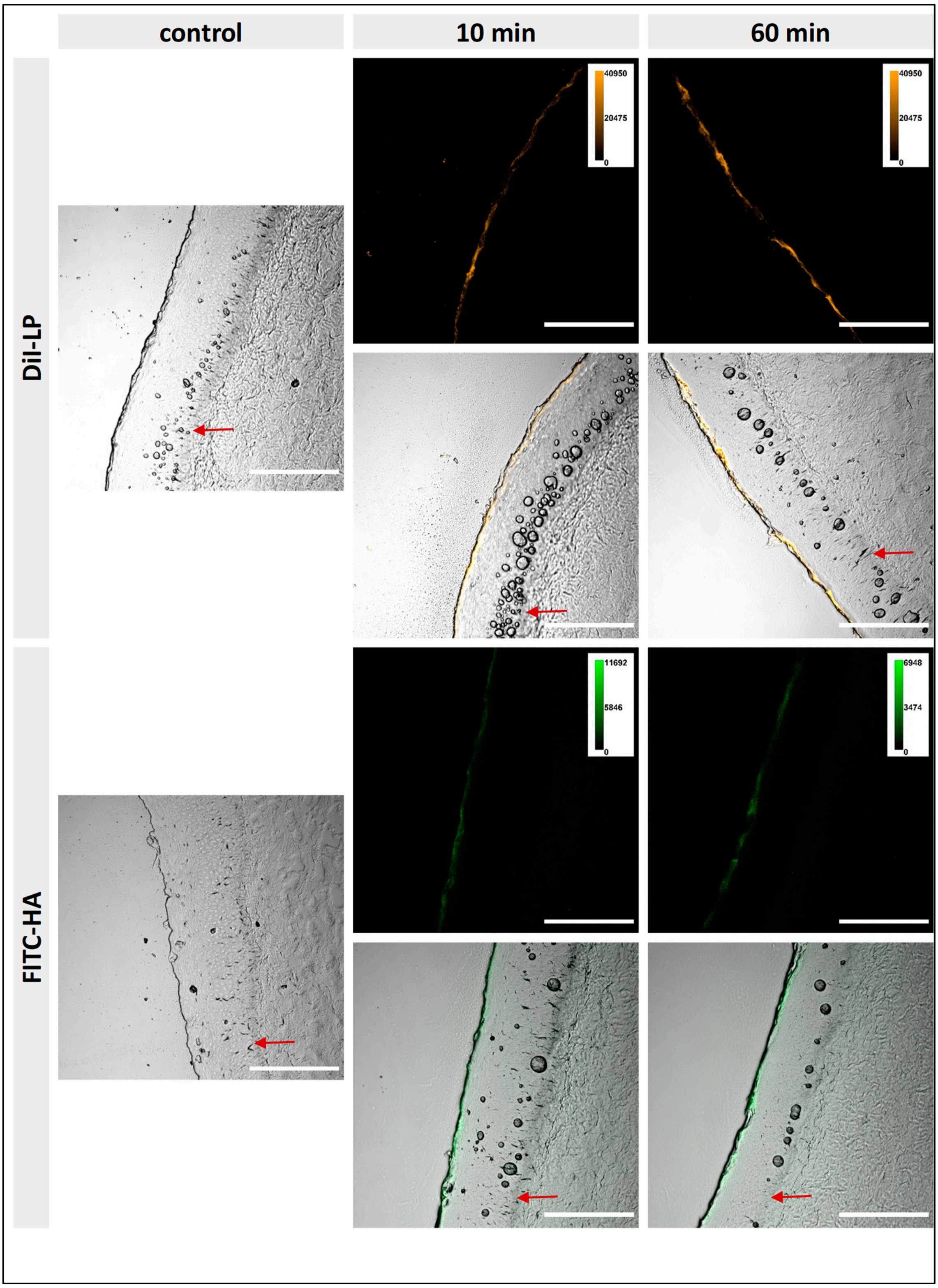

Figure S3. Confocal Laser Scanning Microscopy. Cross sections of blank sheep cornea in transmission light and at excitation with 543,5 nm and 488 nm laser (laser scan and layered image of transmission light and laser scan) for DiI (yellow) and FITC (green) respectively, after incubation with NEOVIS® TOTAL multi (DiI-LP), NEOVIS® TOTAL multi (FITC-HA) at 10 and 60 min. The red indicators point to the basal membrane of the corneal epithelium. Apical direction points left. Scalebar is 200 µm.
